# Characterization of Left Ventricular Non-Compaction Cardiomyopathy

**DOI:** 10.3390/jcm9082524

**Published:** 2020-08-05

**Authors:** Rebeca Lorca, María Martín, Isaac Pascual, Aurora Astudillo, Beatriz Díaz Molina, Helena Cigarrán, Elías Cuesta-Llavona, Pablo Avanzas, José Julían Rodríguez Reguero, Eliecer Coto, César Morís, Juan Gómez

**Affiliations:** 1 Unidad de Referencia de Cardiopatías Familiares-HUCA, Área del Corazón y Departamento de Genética Molecular, Hospital Universitario Central Asturias, 33014 Oviedo, Spain; lorcarebeca@gmail.com (R.L.); mmartinf7@hotmail.com (M.M.); beadimo@gmail.com (B.D.M.); eliascllavona@gmail.com (E.C.-L.); avanzas@gmail.com (P.A.); josejucasa@yahoo.es (J.J.R.R.); eliecer.coto@sespa.es (E.C.); cesarmoris@gmail.com (C.M.); uo167835@uniovi.es (J.G.); 2 Instituto de Investigación Sanitaria del Principado de Asturias, ISPA, 33014 Oviedo, Spain; 3 Faculty of Medicine, University of Oviedo, 33014 Oviedo, Spain; auroastudillo@gmail.com; 4 Anatomía Patológica, Hospital Universitario Central Asturias, 33014 Oviedo, Spain; 5 Servicio de Radiodiagnóstico, Hospital Universitario Central Asturias, 33014 Oviedo, Spain; hcigarran@icloud.com

**Keywords:** left ventricle non-compaction cardiomyopathy, non-ischemic cardiomyopathy, genetics, cardiac magnetic resonance

## Abstract

Left ventricle non-compaction cardiomyopathy (LVNC) has gained great interest in recent years, being one of the most controversial cardiomyopathies. There are several open debates, not only about its genetic heterogeneity, or about the possibility to be an acquired cardiomyopathy, but also about its possible overdiagnosis based on imaging techniques. In order to better understand this entity, we identified 38 LVNC patients diagnosed by cardiac MRI (CMRI) or anatomopathological study that could underwent NGS-sequencing and clinical study. Anatomopathological exam was performed in eight available LVNC hearts. The genetic yield was 34.2%. Patients with negative genetic testing had better left ventricular ejection fraction (LVEF) or it showed a tendency to improve in follow-up, and a possible trigger factor for LVNC was identified in 1/3 of them. Nonetheless, cerebrovascular accidents occurred in similar proportions in both groups. We conclude that in LVNC there seem to be different ways to achieve the same final phenotype. Genetic testing has a good genetic yield and provides valuable information. LVNC without an underlying genetic cause may have a better prognosis in terms of LVEF evolution. However, anticoagulation to prevent cerebrovascular accident (CVA) should be carefully evaluated in all patients. Larger series with pathologic examination are needed to help better understand this entity.

## 1. Introduction

Left ventricular (LV) non-compaction (LVNC) is characterized by prominent myocardial trabeculations in a thick, non-compacted layer adjacent to a thin compacted layer. LVNC is the most recently categorized cardiomyopathy, and probably the most controversial one, without available clinical guidelines. The American Heart Association classified LVNC as a distinct primary cardiomyopathy with a genetic aetiology [1]. However, it is considered an unclassified cardiomyopathy according to the European Society of Cardiology (ESC) [2] or the World Health Organization [3].

LVNC had historically been categorized as congenital condition, secondary to a failure of the compaction process during embryonic cardiac development [4,5,6,7,8]. However, recent data proposed additional etiopathogenic mechanisms, including acquired forms of LVNC secondary to overloading conditions [4,9,10,11]. Therefore, there has been a classical division between isolated LVNC [12,13] and LVNC associated with significant congenital heart defects (CHD) [14,15,16]. In fact, according to Jenni et al. [17], the absence of coexisting cardiac anomalies was mandatory to diagnose LVNC [17]. Nevertheless, it was also suggested that both could actually be co-occurring [18].

Agreement between the three most commonly cited transthoracic echocardiogram (TTE) diagnostic criteria, described by Chin et al. [19], Jenni et al. [17], and Stöllberger et al. [20] is known to be poor [21]. Moreover, there is a raising concern about sensitivity and specificity of TTE criteria, and whether, in fact, LVNC may be overdiagnosed [22,23]. In this scenario, cardiac MRI (CMRI) offers a high spatial resolution, and is becoming more and more used in LVNC evaluation, displacing TTE [24]. However, CMRI also raises concerns about overdiagnosis [25,26,27]. According to diagnostic criteria by Petersen et al., LVNC can be diagnosed if the ratio of non-compacted to compacted myocardium is >2.3 at end-diastole. [28]. Thus, LVNC is commonly associated with other overlapping cardiomyopathies phenotypes. In fact, intrafamilial phenotypic variability, including LVNC, hypertrophic cardiomyopathy (HCM), and dilated cardiomyopathy (DCM), may suggest that these cardiomyopathies could be part of a broader cardiomyopathy spectrum [29,30,31]. In this context, genetics play an important role. From 17% to up to a 50% of patients with LVNC have a relative with another primary cardiomyopathy [32,33,34]. However, due to all the controversy around LVNC and the limitations in assigning its clinical diagnosis based on imaging criteria, strong genetic causal relationships have been harder to establish compared to those in HCM [31,35]. Nowadays, achieving a reliable genetic variants interpretation remains a real challenge and it is likely that some previously interpreted as pathogenic variants [36] in LVNC would need to be reclassified, based on current evidence and new criteria [18,37,38,39].

Moreover, the yield of genetic testing in LVNC varies from 9% to 41%, depending on patient selection and the number of genes screened [18,29,40,41]. Due to small cohort sizes, little is known about LVNC genotype-phenotype correlations. What is more, contrary to HCM guidelines [42], some authors did not support general genetic screening in all patients with LVNC [35].

On the other hand, although a gold-standard diagnostic technique for LVNC is missing [43], anatomopathological examination (APE) could be considered as such. In fact, only three APE cases were enough to support Chin TTE diagnostic criteria [19] and seven for Jenni´s criteria [17]. However, again contrary to HCM, LVNC histopathological characteristics are poorly known. Burke et al. [4,44] established the anatomopathological LVNC criteria based on 14 cases, and only a few more case-series have been published ever since, mostly focusing on compaction/non-compaction ratio and not going deeper into histopathological features.

The aim of the current investigation was to provide a comprehensive clinical view of LVNC based on genetic and anatomopathological information.

## 2. Methods

### 2.1. Study Population

Adult patients (>21 years old) with LVNC diagnosis were recruited consecutively from a tertiary hospital from Spain, referral for cardiogenetics. Due to the controversy about the diagnostic criteria of LVNC, only patients diagnosed with LVNC, either by Petersen CMRI criteria, or Burke APE criteria, were included. Reports from 824 CMRI (from 2007 to 2015) and 89 transplanted hearts (from 2009 to 2015) were reviewed. At this step, LVNC was considered irrespective of its co-occurrence with other primary cardiomyopathies.

According to these criteria, 43 consecutive patients with LVNC diagnosis, either from CMRI criteria (from 2007 to 2015), or form APE criteria (from 2009 to 2015) were identified. Next-generation sequencing was performed in all patients who met the inclusion criteria (excluding significant CHD) and were still alive at the time this study was performed. Three patients could not be included in the clinical study due to decease without genetic testing available. Two patients with LVNC associated with significant CHD (that could induce significant hemodynamic changes) were excluded. Therefore, clinical and genetic study was available for the remaining 38 alive patients with LVNC.

Apart from that, histopathological exam was performed in all available LVNC hearts, including those patients who had died without the possibility of a genetic test.

### 2.2. Clinical Evaluation

A retrospective medical record review of the recruited individuals evaluated was performed. Probands and available family members studied were evaluated by history taking, physical examination, 12-lead electrocardiography, 24-h Holter monitoring, TTE and CMRI or APE. Left ventricular ejection fraction (LVEF) evolution will be categorized into normal, slightly depressed (LVEF <55%), moderately (LVEF 45–35%) depressed, or severely depressed (LVEF <35%).

Possible trigger factors for LVNC (overloading conditions like pregnancy, anaemia o fistula as well as high intensity sport activity) were specifically investigated. Available relatives were screened with the same protocol.

### 2.3. Genetic Testing

Genetic screening was carried out with DNA samples from the 38 LVNC recruited patients. All of them were NGS sequenced for a gene panel including *MYBPC3*, *MYH7*, *TNNI3*, *TNNT2*, *TPM1*, *TNNC*, *MYL1*, *MYL2*, *ACTC1*, *FLNC*, *MIB1*, *TAZ*, *LDB3*, *DTNA*, *HCN4*, *RYR2*, *LMNA*, *NKX2-5*, *MYH6*, *PRDM16*, *ACTN2*, *DMD*, *DNAJC19*, *FHL1*, *PLN*, and *TTN* genes by Ion Torrent semiconductor chip technology in a Ion GeneStudio S5 Sequencer (Thermo Fisher Scientific, Waltham, MA, USA), according to previously described protocols [45,46]. Overall coverage of the gene panel was >95% (Supplementary Table S1). Variant Caller v5 software was used to variant identification (Thermo Fisher Scientific, Waltham, MA, USA). Ion Reporter (Thermo Fisher Scientific, Waltham, MA, USA) and HD Genome One (DREAMgenics S.L., Oviedo, Spain) software were used for variant annotation, including population, functional, disease-related, and in silico predictive algorithms databases.

Data acquisition and analysis was performed in compliance with protocols evaluated by the Ethical Local Committee of the Hospital Universitario Central de Asturias (No. 2020.224). Written informed consent was obtained from all 38 participants, prior to genetic study.

Interpretation of all gene variants with an allele frequency <0.01 was based the American College of Medical Genetics and Genomics (ACMG-AMP) 2015 Standards and Guidelines [37,47,48]. All genetic variants identified in this cohort were reviewed by two biologists and two cardiologists trained in cardiogenetics. Results provided will be divided in 3 groups: (1) pathogenic (P) or likely pathogenic (LP) variants carriers; (2) negative genetic result (benign or likely benign variants); (3) carriers of variants of uncertain significance (VUS). If a P or LP variant was identified direct Sanger sequencing was performed for family screening.

### 2.4. Anatomopathological Exam

All 89 available hearts between 2009 and 2015 from our tertiary referral hospital with a heart transplant program were evaluated. Moreover, a patient diagnosed of LVNC by CMR was also transplanted. APE found eight hearts that fulfilled APE criteria for LVNC: six patients with isolated LVNC, one with congenital heart disease associated and one with concomitant three vessels ischemic disease.

The examination was performed by an experienced pathologist expert in the field, based on the LVNC anatomopathological criteria from Burke et al. [4,44]. Firstly, a macroscopic examination was performed. All hearts were systematically inspected, measured, weighted, and coronary sections were performed. They were examined for pathological changes in the four chambers, septum, pericardium, endocardium, and coronary arteries. Multiple samples were obtained, fixed in formaldehyde, paraffin embedded and stained with haematoxylin/eosin. Macroscopic findings were confirmed in microscopic sections. A minimum of three thin sections from each ventricle and two additionally from the septal area were obtained from paraffin blocks. The macroscopic thickness was measured on the coronary sections of explanted hearts. We selected for microscopy the same area where the macroscopic measurement was performed, and then confirmed the measurements. Compaction and non-compaction wall thickness was measured in coronal macroscopic cuts and ratios were calculated and confirmed in haematoxylin/eosin samples. Histopathological exam was performed, studying fibrosis, inflammation, and cardiomyocytes’ hypertrophy. All sections were stained with Haematoxylin-eosin, Masson trichrome, and Periodic Acid-Schiff (PAS) reaction. Fibre diameter measurements were performed only where the section produced a longitudinal view of cardiomyocytes. The measurement of each diameter was made at the nucleus height, and on a minimum of 50 fibres randomly selected. The nuclear size was measured systematically on longitudinal thinnest axis of nuclei, and over a minimum of 50, randomly selected. We used a Nikon microscope (Tokyo, Japan) with digital camera DS-FI2, and software—Nikon NIS D Elements (Tokyo, Japan), where annotations and measurements were registered. We performed all measurements with the same Nikon planacromatic objective size, using a scale provided from the software programme for each size of lens. The quantification was repeated twice in different journeys and performed by a pathologist and a technician. All the observations and results were reviewed by two professional cardiologists and pathologists.

### 2.5. Statistical Analysis

Statistical analyses were performed with SPSS v.19 (SPSS Inc., Chicago, IL, USA). Descriptive data for continuous variables are presented as mean + SD and as frequencies or percentages for categorical variables. The Chi-square test or Fisher exact test were used to compare frequencies, whereas differences in continuous variables were evaluated with either the Student *t* test or Mann–Whitney *U* test. *p* < 0.05 was considered to be significant.

## 3. Results

### 3.1. Study Population with Genetic and Clinical Evaluation

**Figure 1:**
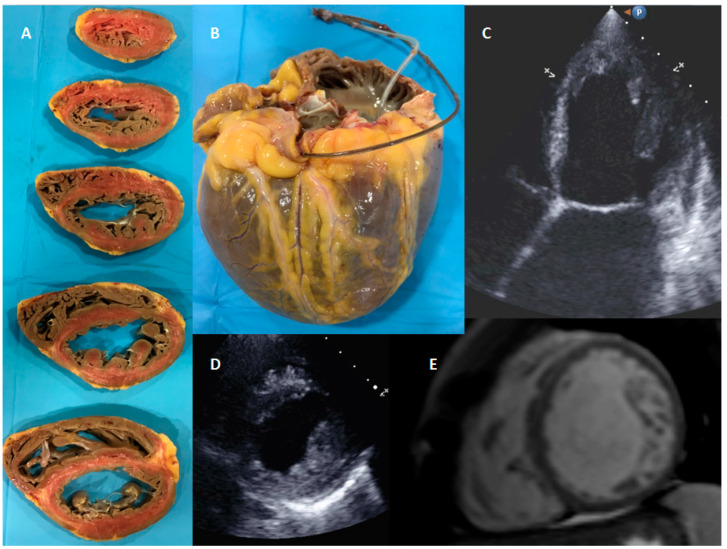
Transplanted patient with isolated left ventricle non-compaction cardiomyopathy diagnosed by CMRI and confirmed by anatomopathological examination. Panel (**A**) Transversal slides of the explanted heart. (**B**) Spheroidal shape of the transplanted heart. (**C**) Apical 4 chamber view in TTE. (**D**) Parasternal short axis in TTE. (**E**) LVNC in CMRI.

A total of 38 isolated LVNC patients diagnosed by CMRI, APE, or both (Figure 1), were evaluated. Results of genetic evaluation are presented in Table 1.

**Figure 2:**
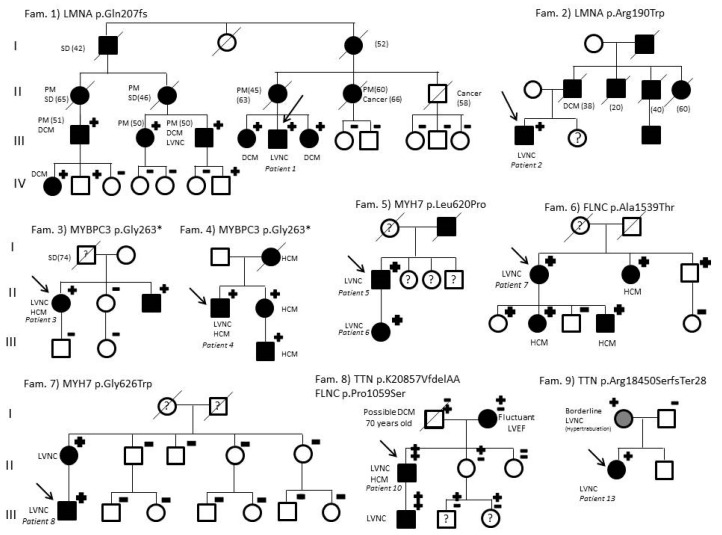
Pedigree of families with LVNC. Fam., family; SD, sudden death; PM, pacemaker; LVNC, left ventricular non-compaction; DCM, dilated cardiomyopathy: HCM, hypertrophic cardiomyopathy. Symbols denote sex and disease status: +, carriers; −, non-carriers; without sign, not studied; box, male; circle, female; darkened, phenotype of hypertrophic cardiomyopathy; symbol clear, unaffected; ?, unknown phenotype; slashed, deceased; without sign, not genetically studied; arrow, proband. Age of deceased or PM implantation in brackets.

A P/LP variant was found in 13 patients (patients 1–13, group 1), meaning a genetic yield of 34.2%. Genetic testing was negative (no relevant genetic variants were identified) in patients 18 to 38 (group 2). Another five patients were carriers of VUS and, therefore, considered separately (patients 14–17, group 3). Family screening was performed in all available relatives, supporting variants segregation criteria in P/LP variants (Figure 2).

Principal clinical characteristics of patients with isolated LVNC are summarized in Table 2. Mean age was 49.4 ± 13.9 SD and 65% of patients were men. Median follow-up of patients was 9.5 years ± 5 SD. Most patients were referred to cardiology due to symptoms, especially due to dyspnoea or other heart failure secondary symptoms. If left ventricular dysfunction was present, optimal medical treatment was given in all patients. LVNC was correctly suspected in fist echocardiogram only in 55% of patients. A cerebrovascular accident (CVA) occurred in 18.4% of patients. In fact, neurological study was the reason for referral in four patients (10.8%). Atrial fibrillation (AF) was identified in only four of the seven patients with ACV. Two patients had suffered CVA with a normal LVEF and without documented AF. What is more, one of them had a recurrent CVA despite and international normalized ratio (INR) of 2.7. Possible trigger factors were identified in 18.4% of patients, most of them due to high intensity sport activity and one of them due to a high flow arteriovenous fistula. Family history of cardiomyopathy was present in 31.5% and 13.15% of patients required heart transplantation.

With genetic screening, up to 22 relatives with P/LP variant carriers and 27 non-carriers relatives were identified. Intrafamilial phenotypic variability was frequently found. As expected, in LMNA families, DCM phenotype was present and HCM in those with sarcomeric pathogenic variants (Figure 2). Moreover, a fluctuant LVEF was found in a relative with previous history of a TTN LP variant carrier (Fam. 8, Figure 2). Clinical and genetic screening for suspicious VUS variants was also performed. However, information obtained was not considered strong enough yet to classify these variants as LP or likely benign variants.

**Figure 3:**
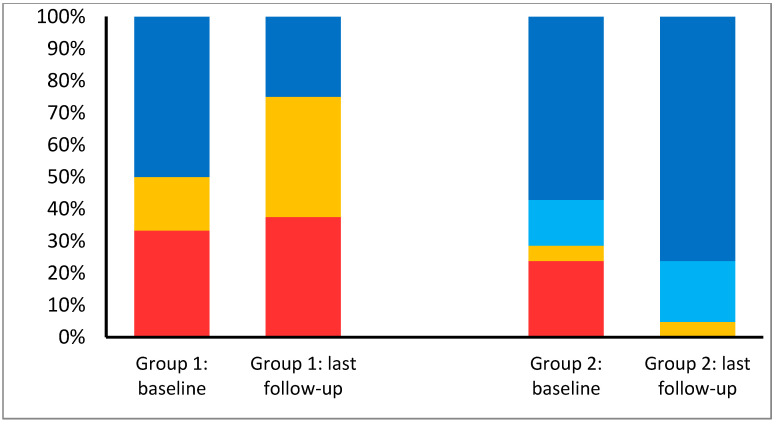
Left ventricle systolic function evolution in follow-up. Group 1, LVNC with pathogenic or likely pathogenic variants; Group 2, LVNC with benign or likely benign variants. Red colour, severely depressed LVEF; yellow, moderately depressed LVEF, bright blue, slightly depressed LVEF; dark blue, normal LVEF.

Main clinical differences between patients with P/LP variants (group 1) and those with negative genetic result, carriers of benign or likely benign variants (group 2) are shown in Table 3. Most patients from group 1 had a known family history of cardiomyopathy. Conversely to group 1, in group 2, a possible trigger factor for LVNC was found in 1/3 of them. Besides, the evolution of LVEF in time showed different patterns between these groups (Figure 3). Initial LVEF in group 2 was better and those impaired a tendency to improve under optimal medical therapy. At baseline, only 57% of them had normal LVEF and 28.6% had a moderate or severe dysfunction. During follow-up, 76.2% of them reached a normal LVEF, being only slightly reduced in 19%. No patients underwent cardiac transplantation or presented severe cardiac dysfunction in follow-up. However, during follow-up, most patients from group 1 (69.2%) presented moderate-severe LV dysfunction and 30.8% underwent heart transplantation.

An internal cardiac defibrilator (ICD) was implanted in 12 patients. Almost half of patients from group 1 had an ICD (46.1%), 50% with at least one appropriate therapy during follow-up. Conversely, only three patients from group 2 (14.3%) received an ICD. What is more, in all three LVEF improved during follow-up and even normalized in two of them.

Apart from that, despite these differences in LVEF, patients suffered cerebrovascular accidents in similar proportions in both groups (23% group 1 vs. 19% group 2, *p* = 0.4). Atrial fibrillation or flutter had been detected in six patients form group 1 and 4 from group 2. However, in both groups, a patient suffered a cerebrovascular accident without previous known arrhythmias.

### 3.2. Anatomopathological Evaluation

Eight LVNC cases that fulfilled APE criteria were evaluated (Figure 1). All patients had been transplanted in final stages of heart failure. Only one patient presented LVNC associated with CHD (heart 6, coarctation of the aorta with severely dilated aortic root and severe aortic insufficiency), and another one presented concomitant ischemic heart disease.

In macroscopic examination, all of them presented a non-compacted layer with prominent myocardial trabeculations, adjacent to a thin compacted layer and prominent myocardial trabeculations. Thickness of both layers was quantified in macroscopy slides and confirmed in haematoxylin/eosin samples, where the measurements were performed. Cellular hypertrophy was evaluated in both layers, and also the presence of fibrosis (Table 4).

**Figure 4:**
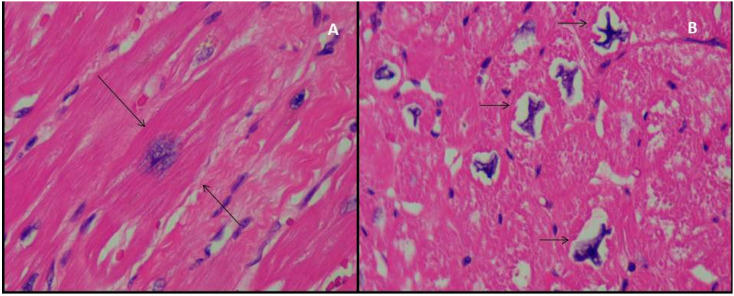
Microscopic haematoxylin/eosin samples (×40) from LVNC explanted hearts. Panel (**A**) myocyte cellular hypertrophy (delimited by arrows); (**B**) deformed nuclear cardiomyocyte shapes.

Cardiomyocytes description deserved special attention. In all analysed cases, their nucleus were enlarged, hyperchromatic and presented irregular striking shapes (Figure 4). Nevertheless, no remarkable abnormal nucleoli were found. Chromatin was, in general soft and without much heterochromatin volume. Cardiomyocyte diameter was enlarged in some of the layers, especially in non-compacted area. In seven cases, neither inflammatory infiltrate, necrosis nor other signs of histological malignancy were found. However, necrosis was identified in one heart, fibrosis in 3 of them, and some areas of slight fat infiltration and some of myocardiosclerosis.

Two patients died after heart transplant, without genetic testing. Out of the six available patients for genetic testing, LP/P variants were found in four of them, a VUS in the fourth one, and only B/LB variants in the other one, with concomitant CHD (heart six, excluded for clinical study). The genetic yield of this small but severely affected cohort of isolated LVNC is 80%.

## 4. Discussion

Over the past few decades, technological advances in genetic sequencing have allowed to perform genetic testing worldwide. The number of genetic variants to analyse has increased massively [49] and so has the complexity of its interpretation. Achieving a reliable classification is crucial [36], especially in controversial entities like LVNC. LVNC is the most recently described cardiomyopathy and broadening the knowledge of its genetics field is an absolute necessity. Genetic yield is really variable depending on the reported series and very few papers have tried to compare LVNC phenotype with or without an identifiable genetic cause. Moreover, genetic variants classification performed before ACMG-AMP criteria [37] should be interpreted with caution. In addition, most studies included LVNC patients diagnosed only based on TTE criteria.

A study performed about 10 years ago, found a genetic “mutation” in 17.5% of their TTE-based cohort [40]. The generic yield improved to 29% in a study of 63 isolated LVNC diagnosed by TTE [50] and to 41% in another TTE based study [29]. However, no differences in clinical phenotypes between positive and negative pathogenic variants’ carriers were found in either of these two studies.

In 2017, an interesting study in children classified variants according to ACMG [37] with a genetic yield of only 9% and, unfortunately, no comparison between carriers and non-carriers were done [18]. Wang et al. also analysed a childhood cohort with a higher genetic yield [38%] describing a poorer prognosis in pathogenic variants carriers (earlier age of onset and lower LVEF) than those without pathogenic variants [41]. Another German study of 68 index LVCN patients diagnosed by TTE reported a 38% genetic yield and described worse clinical outcomes in patients with pathogenic variants in LMNA and RBM20. In their cohort, TTN variants were the most frequent cause for LVNC and they associated TTN truncating variants with LVNC phenotype [51].

In our study, genetic testing identified a genetic cause in up to 34.2% of patients, a percentage within the expected ranges according to previously reported series. However, better understanding of VUS may improve this yield. Thanks to genetic screening not only 22 relatives at risk were identified, but also 27 relatives could be discharged. LVNC have been accepted to be a possible inherited condition. Therefore, we believe that genetic screening should be strongly recommended, like in any other kind inherited cardiomyopathy. Moreover, in this entity, genetic testing may be useful not only for family screening, but also to help in differential diagnosis with hypertrabeculation mimicking LVNC. In fact, if genetic testing had only been performed in those with moderate to severe LV dysfunction in follow-up, genetic yield would have improved to 75% (9/12).

The strength of our cohort relies not only in the genetic variants’ classification based on ACMG-AMP guidelines [37], but also on the patient’s selection to achieve a LNVC cohort with a solid diagnosis. Carriers of VUS (group 3) were not included in any comparison group, as its classification may easily change into LP or LB as genetic knowledge improves. However, interesting differences between carriers of P/LP variants (group 1) and non-carriers (group 2) were found. Family history of cardiomyopathies is mainly found in group 1, and possible trigger factors were only identified in group 2. In fact, trigger factors may explain the hypertrabeculation in one in every three patients from group 2. However, the most stunning finding was the LVEF evolution in follow-up (Figure 3). LVEF showed a tendency to improve in time in group 2, contrary to the tendency to worsen in group 1. Although these results should be interpreted with caution, genetic results could represent a predictor factor for LVEF evolution, especially if a trigger factor had been identified. In our cohort, this information could have been useful, for example, to delay the ICD implant decision in patients from group 2, whose LVEF improved with optimal medical therapy. Besides, all carries of TTN variants presented a fluctuant LVEF. On the other hand, CVAs are present in the same proportion in both groups, highlighting the importance of emboli risk assessment in all LVNC phenotypes.

We believe that our data reinforce the hypothesis that there are different ways to reach the same final phenotype, whether as congenital heart defect or as an inherited cardiomyopathy or as an acquired one. Although in this study we included LVNC patients based on the same criteria, they had different etiologies. It seems obvious that patients from group 1 have an inherited kind of cardiomyopathy. However, the presence of trigger factors in 1/3 of patients from group 2 support the hypothesis that LVNC phenotype could also be an acquired cardiomyopathy [4,9,10,11]. On the basis of an apparently normal heart with an underlying predisposition, the developing of hypertrabeculation leading to LVNC phenotype may (or may not) start to manifest at a certain age. Although prognostic differences in these different scenarios may be found (highlighting the role of genetic testing and LVEF evolution), clinicians should be aware of possible related complications like CVA in all LVNC phenotypes.

In addition, our data also supports the theory that LVNC could actually been underdiagnosed previously, even in autopsies [13,21,44,52]. In our series, TTE misdiagnosed LVNC in the first place in up to 45% of LVNC cohort. No wonder why the diagnostic based on imaging criteria is still a real challenge and an ongoing debate.

Furthermore, APE has played a critical role in helping to describe and classify LVNC as a novel cardiomyopathy and to settle its diagnostic criteria [17,19]. Nevertheless, contrary to HCM, there are no large case series available [44,53] and deep histopathological characterization is missing. HCM is known for its disarray, hypertrophy of myocytes and possible fibrosis [54]. This hypertrophy of myocytes is maximal at subendocardium [55,56] and nuclei can be enlarged, presenting nuclear pleomorphism and hyperchromasia [54]. In LVNC, APE data have been focused in compaction/non-compaction layers description and quantification [4,44], neglecting meticulous histopathological description [4,57]. Fibrosis areas considered secondary to ischemia due to microvascular dysfunction are described in some studies [58,59,60]. Burke et al. [4,44] did not found any difference between isolated LVNC and associated with CHD. Although Jenni et al. [17] claimed that no disarray was present, a recent transplantation series found it in one LVNC patient [59]. Proper myocyte description beyond isolated cases is also missing [59,61,62], and no genetic data are reported in any series. For this reason, the APE description of our sample, although small, is important. According to previous data, fibrosis and one case of necrosis were identified, but no inflammatory pattern. However, the most important finding was the cellular hypertrophy of myocytes, present in all the studied hearts. Moreover, its distribution is not uniform, being more pronounced in the non-compacted layer. However, the most striking feature was the irregular nuclear shape (Figure 4). In addition, the available genetic data, with a good yield in these patients with severe phenotype of LVNC, are really interesting. Although the described histopathological findings are present in the eight hearts, two of them intriguingly present a LP variant in a gene that encodes a component of the nuclear lamina, which determines nuclear shape and size.

## 5. Limitations

Family screening was not available for all relatives. All genetic variants identified in this cohort were reviewed by two biologists and two cardiologists trained in cardiogenetics, according to current published guidelines and available data [37]. Despite these efforts, some variants may be reclassified as additional data become available. Family screening for TTN variants from patients 11 and 12 are still pending due to the COVID-19 pandemic. The genetic testing yield may also improve as gene panels continue to expand and better classification of VUS is achieved. Further investigation in genetics and histopathological exam, expanding the series number, is definitely necessary to draw further conclusions.

## 6. Conclusions

There seem to be different ways to achieve the same final phenotype: LVNC. As genetic testing in LVNC has a good genetic yield and provides valuable information, it should be recommended for all LVNC patients. LVNC without an underlying genetic cause may have a better prognosis in terms of LVEF evolution in time. However, anticoagulation to prevent CVA should be carefully evaluated in all patients. Larger series with pathologic examination are needed to help better understand this entity.

## Figures and Tables

**Table 1:** Remarkable identified genetic variants: Pathogenic (P), likely pathogenic (LP) variants, or variants of uncertain significance (VUS), classified according to American College of Medical Genetics and Genomics (ACMG) [37], in our LVNC cohort.

Patient	GENE	hg38	NM	PROTEIN	cDNA	FUNCTION	GnomAD Exomes Frequency	HCMG-AMP
1	LMNA	chr1:156134508	NM_170707	p.Gln207ArgfsTer273	c.619delC	Truncating	–	P
2	LMNA	chr1:156134457	NM_170707	p.Arg190Trp	c.568C > T	missense	–	LP
3	MYBPC3	chr11:47347891	NM_000256	p.Gly263Ter	c.787G > T	Truncating	–	P
4	MYBPC3	chr11:47347891	NM_000256	p.Gly263Ter	c.787G > T	Truncating	–	P
5	MYH7	chr14:23427614	NM_000257	p.Leu620Pro	c.1859T > C	missense	–	LP
6	MYH7	chr14:23427614	NM_000257	p.Leu620Pro	c.1859T > C	missense	–	LP
7	FLNC	chr7:128848595	NM_001458	p.Ala1539Thr	c.4615G > A	missense	–	LP
8	MYH7	chr14:23427597	NM_000257	p.Gly626Trp	c.1876G > T	missense	–	LP
9	TTN	chr2: 178553135	NM_003319	p.Lys20857ValfsTer7	c.62569_62570delAA	Truncating	–	LP
9	FLNC	chr7: 28844249	NM_001458	p.Pro1059Ser	c.3175C > T	missense	–	VUS
10	MYH7	chr14:23430954	NM_000257	p.Arg281Lys	c.842G > C	missense	–	LP
11	TTN	chr2: 78557876	NM_003319	p.Glu20095Ter	c.60283G > T	Truncating	–	LP
12	TTN	chr2:178546323	NM_003319	p.Arg22605Ter	c.67813C > T	Truncating	0.00000402	LP
13	TTN	chr2:178563588	NM_003319	p.Arg18450SerfsTer28	c.55346_55349dupTTAG	Truncating	–	LP
13	ACTN2	chr1: 236717925	NM_001103	p.Asp65Ala	c.194A > C	missense	–	VUS
14	MYH6	chr14:23862208	NM_002471.3	p.Arg1055Gln	c.3164G > A	missense	0.000123	VUS
15	MYH7	chr14: 23424965	NM_000257	p.Pro828Leu	c.2483C > T	missense	–	VUS
16	RBM20	chr10:110780815	NM_001134363	p.Leu69Pro	c.206T > C	missense	–	VUS
17	TTN	chr2: 178775139	NM_003319	p.Met2145GlyfsTer4	c.6433_6434delAT	Truncating	–	VUS

**Table 2:** Clinical characteristics of LVNC patients. CVA, cerebrovascular accident, EKG, electrocardiography; TTE, transthoracic echocardiogram; Tx, transplanted; FH, family history; LVEF, left ventricular ejection fraction (0, normal; 1, mild depressed, 2; moderate; 3, severely depressed).

Patient	Gender	Genetics	LVNC Suspicion in TTE	Reason for Referral	CVA	Tx	FH	Trigger Factors	LVEF Evolution
1	Male	P/LP	Yes	Dyspnoea/arrhythmia	no	yes	yes	no	2–3
2	Male	P/LP	No	EKG	no	yes	yes	no	2–3
3	Female	P/LP	No	CVA	Yes	no	yes	no	0–2
4	Male	P/LP	Yes	Heart murmur	Yes	no	yes	no	0–3
5	Male	P/LP	No	Syncope	no	no	yes	no	0–1–0
6	Female	P/LP	Yes	Family screening	no	no	yes	no	0
7	Female	P/LP	Yes	Dyspnoea/palpitations	no	yes	yes	no	0–3
8	Male	P/LP	Yes	Heart failure	no	no	yes	no	3–2
9	Male	P/LP	No	unknown	no	yes	no	no	3–2–3
10	Female	P/LP	No	Cardiogenic Shock	Yes	no	no	no	0
11	Male	P/LP	Yes	Cardiogenic Shock	no	no	no	no	3–1–3
12	Male	P/LP	Yes	unknown	no	no	no	no	3–2
13	Female	P/LP	No	Dyspnoea	no	no	no	no	0
14	Female	VUS	No	Dyspnoea	no	Yes	yes	no	2–3
15	Female	VUS	Yes	Palpitations/Syncope	no	no	no	no	0
16	Male	VUS	No	EKG	no	no	no	no	2–3
17	Male	VUS	No	Ischemic heart disease	no	no	no	no	0–3–1
18	Female	Negative	No	Dyspnoea	no	no	no	yes	1–0
19	Female	Negative	Yes	Vagal syncope	no	no	yes	yes	0
20	Male	Negative	No	Dyspnoea	no	no	no	no	3–1
21	Male	Negative	Yes	Family screening	no	no	yes	no	3–1
22	Male	Negative	No	Neurological study	Yes	no	no	no	3–0
23	Female	Negative	Yes	Dyspnoea	Yes	no	no	no	2–0
24	Male	Negative	Yes	Heart failure	no	no	no	no	3–0
25	Female	Negative	No	Heart failure	no	no	no	no	3–0
26	Male	Negative	Yes	Heart failure	no	no	no	no	3–2
27	Male	Negative	No	EKG	no	no	no	no	1–2–1
28	Male	Negative	No	EKG	no	no	no	yes	0
29	Male	Negative	Yes	Palpitations	no	no	no	yes	0
30	Male	Negative	No	EKG	no	no	yes	yes	0–1
31	Female	Negative	Yes	CVA	Yes	no	no	no	0
32	Male	Negative	Yes	Heart murmur	no	no	no	no	0
33	Male	Negative	Yes	CVA	Yes	no	no	no	0
34	Male	Negative	Yes	EKG	no	no	no	no	0
35	Male	Negative	Yes	Syncope	no	no	no	no	0
36	Male	Negative	Yes	unknown	no	no	no	yes	0
37	Female	Negative	Yes	Palpitations	no	no	no	no	0
38	Male	Negative	No	EKG	no	no	no	yes	0

**Table 3:** Clinical characteristics of carriers of P/LP variants (group 1) and patients with negative genetic result, carriers of benign or likely benign variants (group 2).

	Group 1	Group 2
% Men	61.5%	75%
Possible trigger factors for LVNC	0%	33.3%
Family history of cardiomyopathy	61.5%	15%
LVEF evolution	Tendency to worsen	Normal/Tendency to improve
Heart transplantation	30.%	0%

**Table 4:** Histopathological characteristics of hearts with LVNC diagnosis. NC, non-compaction; C, compaction; LV, left ventricle. Cellular hypertrophy: 0 = none; 1 = mild; 2 = moderate; 3 = severe.

Heart	NC Thickness	C Thickness	LV wall Thickness	NC/C	Fibrosis	NC Cellular Hypertrophy	C Cellular Hypertrophy	Genetic Variants
1	16	8	23	2	yes	3	3	LMNA p.Gln207fs
2	16	5	21	3.2	no	3	2	LMNA p.Arg190Trp
3	16	7	23	2.3	yes	Not valuable	1	FLNC p.Ala1539Thr
4	11	4	15	2.7	no	3	2	TTN p.K20857VfsdelAA FLNC p.Pro1059Ser
5	17	5	22	3.4	no	3	2	MYH6 R1055Q
6	20	5	25	4	no	3	2	Negative
7	14	3	17	4.6	no	3	2	unavailable
8	17	7	24	2.4	yes	2	2	unavailable

## Supplementary Materials

The following are available online at https://www.mdpi.com/2077-0383/9/8/2524/s1, Table S1: Overall coverage of the gene panel.

## References

[1] Maron B.J., Towbin J.A., Thiene G., Antzelevitch C., Corrado D., Arnett D., Moss A.J., Seidman C.E., Young J.B. (2006). Contemporary definitions and classification of the cardiomyopathies: An American Heart Association Scientific Statement from the Council on Clinical Cardiology, Heart Failure and Transplantation Committee; Quality of Care and Outcomes Research and Functional Genomics and Translational Biology Interdisciplinary Working Groups; and Council on Epidemiology and Prevention. Circulation.

[2] Elliott P., Andersson B., Arbustini E., Bilinska Z., Cecchi F., Charron P., Dubourg O., Kühl U., Maisch B., McKenna W.J. (2008). Classification of the cardiomyopathies: A position statement from the European Society Of Cardiology Working Group on Myocardial and Pericardial Diseases. Eur. Heart J..

[3] Richardson P., McKenna W., Bristow M., Maisch B., Mautner B., O’Connell J., Olsen E., Thiene G., Goodwin J., Gyarfas I. (1996). Report of the 1995 World Health Organization/International Society and Federation of Cardiology Task Force on the Definition and Classification of cardiomyopathies. Circulation.

[4] Arbustini E., Weidemann F., Hall J.L. (2014). Left ventricular noncompaction: A distinct cardiomyopathy or a trait shared by different cardiac diseases?. J. Am. Coll. Cardiol..

[5] Liu J., Bressan M., Hassel D., Huisken J., Staudt D., Kikuchi K., Poss K.D., Mikawa T., Stainier D.Y. (2010). A dual role for ErbB2 signaling in cardiac trabeculation. Development.

[6] Gupta V., Poss K.D. (2012). Clonally dominant cardiomyocytes direct heart morphogenesis. Nature.

[7] Risebro C.A., Riley P.R. (2006). Formation of the ventricles. Sci. World J..

[8] Sedmera D., Thompson R.P. (2011). Myocyte proliferation in the developing heart. Dev. Dyn..

[9] Gati S., Papadakis M., Van Niekerk N., Reed M., Yeghen T., Sharma S. (2013). Increased left ventricular trabeculation in individuals with sickle cell anaemia: Physiology or pathology?. Int. J. Cardiol..

[10] Gati S., Papadakis M., Papamichael N.D., Zaidi A., Sheikh N., Reed M., Sharma R., Thilaganathan B., Sharma S. (2014). Reversible de novo left ventricular trabeculations in pregnant women: Implications for the diagnosis of left ventricular noncompaction in low-risk populations. Circulation.

[11] Gati S., Chandra N., Bennett R.L., Reed M., Kervio G., Panoulas V.F., Ghani S., Sheikh N., Zaidi A., Wilson M. (2013). Increased left ventricular trabeculation in highly trained athletes: Do we need more stringent criteria for the diagnosis of left ventricular non-compaction in athletes?. Heart.

[12] Engberding R., Bender F. (1984). Identification of a rare congenital anomaly of the myocardium by two-dimensional echocardiography: Persistence of isolated myocardial sinusoids. Am. J. Cardiol..

[13] Ritter M., Oechslin E., Sutsch G., Attenhofer C., Schneider J., Jenni R. (1997). Isolated noncompaction of the myocardium in adults. Mayo Clin. Proc..

[14] Grant T. (1926). An unusual anomaly of the coronary vessels in the malformed heart of a child. Heart.

[15] Pignatelli R.H., McMahon C.J., Dreyer W.J., Denfield S.W., Price J., Belmont J.W., Craigen W.J., Wu J., El Said H., Bezold L.I. (2003). Clinical characterization of left ventricular noncompaction in children: A relatively common form of cardiomyopathy. Circulation.

[16] Jenni R., Rojas J., Oechslin E. (1999). Isolated noncompaction of the myocardium. N. Engl. J. Med..

[17] Jenni R., Oechslin E., Schneider J., Attenhofer Jost C., Kaufmann P.A. (2001). Echocardiographic and pathoanatomical characteristics of isolated left ventricular non-compaction: A step towards classification as a distinct cardiomyopathy. Heart.

[18] Miller E.M., Hinton R.B., Czosek R., Lorts A., Parrott A., Shikany A.R., Ittenbach R.F., Ware S.M. (2017). Genetic testing in pediatric left ventricular noncompaction. Circ. Cardiovasc. Genet..

[19] Chin T.K., Perloff J.K., Williams R.G., Jue K., Mohrmann R. (1990). Isolated noncompaction of left ventricular myocardium. A study of eight cases. Circulation.

[20] Stollberger C., Gerecke B., Finsterer J., Engberding R. (2013). Refinement of echocardiographic criteria for left ventricular noncompaction. Int. J. Cardiol..

[21] Kohli S.K., Pantazis A.A., Shah J.S., Adeyemi B., Jackson G., McKenna W.J., Sharma S., Elliott P.M. (2008). Diagnosis of left-ventricular non-compaction in patients with left-ventricular systolic dysfunction: Time for a reappraisal of diagnostic criteria?. Eur. Heart J..

[22] Habib G., Charron P., Eicher J.C., Giorgi R., Donal E., Laperche T., Boulmier D., Pascal C., Logeart D., Jondeau G. (2011). Isolated left ventricular non-compaction in adults: Clinical and echocardiographic features in 105 patients. Results from a French registry. Eur. J. Heart Fail..

[23] Niemann M., Stork S., Weidemann F. (2012). Left ventricular noncompaction cardiomyopathy: An overdiagnosed disease. Circulation.

[24] Yoon Y.E., Hong Y.J., Kim H.K., Kim J.A., Na J.O., Yang D.H., Kim Y.J., Choi E.Y. (2014). The Korean Society of Cardiology and the Korean Society of Radiology. 2014 Korean guidelines for appropriate utilization of cardiovascular magnetic resonance imaging: A joint report of the Korean Society of Cardiology and the Korean Society of Radiology. Korean J. Radiol..

[25] Ross S.B., Jones K., Blanch B., Puranik R., McGeechan K., Barratt A., Semsarian C. (2020). A systematic review and meta-analysis of the prevalence of left ventricular non-compaction in adults. Eur. Heart J..

[26] Ross S.B., McGeechan K., Barratt A., Semsarian C. (2019). Overdiagnosis of left ventricular non-compaction in adults: The data tells the story. Eur. Heart J..

[27] Protonotarios A., Elliott P.M. (2019). Left ventricular non-compaction: Have we reached the limits of conventional imaging?. Eur. Heart J..

[28] Petersen S.E., Selvanayagam J.B., Wiesmann F., Robson M.D., Francis J.M., Anderson R.H., Watkins H., Neubauer S. (2005). Left ventricular non-compaction: Insights from cardiovascular magnetic resonance imaging. J. Am. Coll. Cardiol..

[29] Hoedemaekers Y.M., Caliskan K., Michels M., Frohn-Mulder I., van der Smagt J.J., Phefferkorn J.E., Wessels M.W., ten Cate F.J., Sijbrands E.J., Dooijes D. (2010). The importance of genetic counseling, DNA diagnostics, and cardiologic family screening in left ventricular noncompaction cardiomyopathy. Circ. Cardiovasc. Genet..

[30] Lorca R., Martín M., Gómez J., Santamarta E., Morís C., Reguero J.J., Coto E. (2015). Hypertrophic cardiomyopathy and left ventricular non-compaction: Different manifestations of the same cardiomyopathy spectrum?. Int. J. Cardiol..

[31] Van Waning J.I., Caliskan K., Hoedemaekers Y.M., van Spaendonck-Zwarts K.Y., Baas A.F., Boekholdt S.M., van Melle J.P., Teske A.J., Asselbergs F.W., Backx A. (2018). Genetics, clinical features, and long-term outcome of noncompaction cardiomyopathy. J. Am. Coll. Cardiol..

[32] Oechslin E.N., Attenhofer Jost C.H., Rojas J.R., Kaufmann P.A., Jenni R. (2000). Long-term follow-up of 34 adults with isolated left ventricular noncompaction: A distinct cardiomyopathy with poor prognosis. J. Am. Coll. Cardiol..

[33] Weiford B.C., Subbarao V.D., Mulhern K.M. (2004). Noncompaction of the ventricular myocardium. Circulation.

[34] Ichida F., Hamamichi Y., Miyawaki T., Ono Y., Kamiya T., Akagi T., Hamada H., Hirose O., Isobe T., Yamada K. (1999). Clinical features of isolated noncompaction of the ventricular myocardium: Long-term clinical course, hemodynamic properties, and genetic background. J. Am. Coll. Cardiol..

[35] Finsterer J., Stollberger C., Towbin J.A. (2017). Left ventricular noncompaction cardiomyopathy: Cardiac, neuromuscular, and genetic factors. Nat. Rev. Cardiol..

[36] Walsh R., Thomson K.L., Ware J.S., Funke B.H., Woodley J., McGuire K.J., Mazzarotto F., Blair E., Seller A., Taylor J. (2017). Reassessment of Mendelian gene pathogenicity using 7,855 cardiomyopathy cases and 60,706 reference samples. Genet. Med..

[37] Richards S., Aziz N., Bale S., Bick D., Das S., Gastier-Foster J., Grody W.W., Hegde M., Lyon E., Spector E. (2015). Standards and guidelines for the interpretation of sequence variants: A joint consensus recommendation of the American College of Medical Genetics and Genomics and the Association for Molecular Pathology. Genet. Med..

[38] Finsterer J., Stollberger C. (2014). Are RYR2 exon-3 deletions truly causative for non-compaction?. Europace.

[39] Finsterer J., Zarrouk-Mahjoub S. (2015). Lamin A/C mutations do not cause left ventricular hypertrabeculation/noncompaction. Tex. Heart Inst. J..

[40] Klaassen S., Probst S., Oechslin E., Gerull B., Krings G., Schuler P., Greutmann M., Hürlimann D., Yegitbasi M., Pons L. (2008). Mutations in sarcomere protein genes in left ventricular noncompaction. Circulation.

[41] Wang C., Hata Y., Hirono K., Takasaki A., Ozawa S.W., Nakaoka H., Saito K., Miyao N., Okabe M., Ibuki K. (2017). A wide and specific spectrum of genetic variants and genotype-phenotype correlations revealed by next-generation sequencing in patients with left ventricular noncompaction. J. Am. Heart Assoc..

[42] Elliott P.M., Anastasakis A., Borger M.A., Borggrefe M., Cecchi F., Charron P., Hagege A.A., Lafont A., Limongelli G., Task Force Members (2014). 2014 ESC Guidelines on diagnosis and management of hypertrophic cardiomyopathy: The Task Force for the Diagnosis and Management of Hypertrophic Cardiomyopathy of the European Society of Cardiology (ESC). Eur. Heart J..

[43] Yin L. (2014). Non-compact cardiomyopathy or ventricular non-compact syndrome?. J. Cardiovasc. Ultrasound..

[44] Burke A., Mont E., Kutys R., Virmani R. (2005). Left ventricular noncompaction: A pathological study of 14 cases. Hum. Pathol..

[45] Gómez J., Reguero J.R., Morís C., Martín M., Alvarez V., Alonso B., Iglesias S., Coto E. (2014). Mutation analysis of the main hypertrophic cardiomyopathy genes using multiplex amplification and semiconductor next-generation sequencing. Circ. J..

[46] Gómez J., Lorca R., Reguero J.R., Morís C., Martín M., Tranche S., Alonso B., Iglesias S., Alvarez V., Díaz-Molina B. (2017). Screening of the filamin C gene in a large cohort of hypertrophic cardiomyopathy patients. Circ. Cardiovasc. Genet..

[47] Telenti A., Pierce L.C., Biggs W.H., di Iulio J., Wong E.H., Fabani M.M., Kirkness E.F., Moustafa A., Shah N., Xie C. (2016). Deep sequencing of 10,000 human genomes. Proc. Natl. Acad. Sci. USA.

[48] Jagadeesh K.A., Wenger A.M., Berger M.J., Guturu H., Stenson P.D., Cooper D.N., Bernstein J.A., Bejerano G. (2016). M-CAP eliminates a majority of variants of uncertain significance in clinical exomes at high sensitivity. Nat. Genet..

[49] Sen-Chowdhry S., Jacoby D., Moon J.C., McKenna W.J. (2016). Update on hypertrophic cardiomyopathy and a guide to the guidelines. Nat. Rev. Cardiol..

[50] Probst S., Oechslin E., Schuler P., Greutmann M., Boyé P., Knirsch W., Berger F., Thierfelder L., Jenni R., Klaassen S. (2011). Sarcomere gene mutations in isolated left ventricular noncompaction cardiomyopathy do not predict clinical phenotype. Circ. Cardiovasc. Genet..

[51] Sedaghat-Hamedani F., Haas J., Zhu F., Geier C., Kayvanpour E., Liss M., Lai A., Frese K., Pribe-Wolferts R., Amr A. (2017). Clinical genetics and outcome of left ventricular non-compaction cardiomyopathy. Eur. Heart J..

[52] Rigopoulos A., Rizos I.K., Aggeli C., Kloufetos P., Papacharalampous X., Stefanadis C., Toutouzas P. (2002). Isolated left ventricular noncompaction: An unclassified cardiomyopathy with severe prognosis in adults. Cardiology.

[53] Val-Bernal J.F., Garijo M.F., Rodriguez-Villar D., Val D. (2010). Non-compaction of the ventricular myocardium: A cardiomyopathy in search of a pathoanatomical definition. Histol. Histopathol..

[54] Hughes S.E. (2004). The pathology of hypertrophic cardiomyopathy. Histopathology.

[55] Unverferth D.V., Baker P.B., Pearce L.I., Lautman J., Roberts W.C. (1987). Regional myocyte hypertrophy and increased interstitial myocardial fibrosis in hypertrophic cardiomyopathy. Am. J. Cardiol..

[56] Varnava A.M., Elliott P.M., Sharma S., McKenna W.J., Davies M.J. (2000). Hypertrophic cardiomyopathy: The interrelation of disarray, fibrosis, and small vessel disease. Heart.

[57] Gerger D., Stöllberger C., Grassberger M., Gerecke B., Andresen H., Engberding R., Finsterer J. (2013). Pathomorphologic findings in left ventricular hypertrabeculation/noncompaction of adults in relation to neuromuscular disorders. Int. J. Cardiol..

[58] Finsterer J., Stollberger C., Feichtinger H. (2002). Histological appearance of left ventricular hypertrabeculation/noncompaction. Cardiology.

[59] Ottaviani G., Segura A.M., Rajapreyar I.N., Zhao B., Radovancevic R., Loyalka P., Kar B., Gregoric I., Buja L.M. (2016). Left ventricular noncompaction cardiomyopathy in end-stage heart failure patients undergoing orthotopic heart transplantation. Cardiovasc. Pathol..

[60] Jenni R., Wyss C.A., Oechslin E.N., Kaufmann P.A. (2002). Isolated ventricular noncompaction is associated with coronary microcirculatory dysfunction. J. Am. Coll. Cardiol..

[61] Bleyl S.B., Mumford B.R., Brown-Harrison M.C., Pagotto L.T., Carey J.C., Pysher T.J., Ward K., Chin T.K. (1997). Xq28-linked noncompaction of the left ventricular myocardium: Prenatal diagnosis and pathologic analysis of affected individuals. Am. J. Med. Genet..

[62] Ivan D., Flamm S.D., Abrams J., Kindo M., Heck K., Frazier O.H. (2005). Isolated ventricular non-compaction in adults with idiopathic cardiomyopathy: Cardiac magnetic resonance and pathologic characterization of the anomaly. J. Heart Lung Transplant..

