# Supplementary material for: Characterization of Left Ventricular Non-Compaction Cardiomyopathy
Source: J Clin Med. 2020 Aug 5;9(8):2524. doi: 10.3390/jcm9082524 (PMC7464545; doi:10.3390/jcm9082524)
Supplement: Supplementary file 1 [file jcm-09-02524-s001.pdf]

Table S1. Overall coverage of the gene panel.

Gene	Chr	# Amplicons	Total Bases	Covered Bases	Missed Bases	Overall Coverage
ACTC1	chr15	8	1194	1194	0	100%
ACTN2	chr1	28	3112	3112	0	100%
DMD	chrX	106	12161	12161	0	100%
DNAJC19	chr3	6	411	411	0	100%
DTNA	chr18	29	3795	3795	0	100%
FHL1	chrX	11	1472	1466	6	99.59%
FLNC	chr7	75	8658	8658	0	100%
HCN4	chr15	28	4012	4012	0	100%
LDB3	chr10	24	2668	2664	4	99.85%
LMNA	chr1	18	2369	2369	0	100%
MIB1	chr18	36	4071	4071	0	100%
MYBPC3	chr11	35	4155	4155	0	100%
MYH6	chr14	51	6190	6190	0	100%
MYH7	chr14	45	6188	6188	0	100%
MYL2	chr12	7	571	571	0	100%
MYL3	chr3	6	648	648	0	100%
NKX2-5	chr5	9	1142	1142	0	100%
PLN	chr6	1	169	169	0	100%
PRDM16	chr1	37	4681	4654	27	99.42%
RYR2	chr1	134	15954	15954	0	100%
TAZ	chrX	14	1043	1043	0	100%
TNNC1	chr3	7	546	546	0	100%
TNNI3	chr19	7	702	702	0	100%
TNNT2	chr1	17	1081	1081	0	100%
TPM1	chr15	15	1500	1500	0	100%
TTN	chr2	800	132581	129775	2806	97.88%
